# Structured reporting: a fusion reactor hungry for fuel

**DOI:** 10.1007/s13244-014-0368-7

**Published:** 2014-12-05

**Authors:** Jan M. L. Bosmans, Emanuele Neri, Osman Ratib, Charles E. Kahn Jr.

**Affiliations:** 1 Departments of Radiology and Medical Imaging, Ghent University Hospital and University of Antwerp, Ghent and Antwerp, Flanders, Belgium; 2 Department of Diagnostic and Interventional Radiology, University of Pisa, Pisa, Italy; 3 Department of Medical Imaging and Information Sciences, University Hospital of Geneva, Geneva, Switzerland; 4 Department of Radiology, University of Pennsylvania, Philadelphia, PA USA

## Introduction

Analysis of a serum sample, and whole blood on citrate and EDTA. We compare the results to those of December 20th. We observe a slight rise in blood glucose level, but the value is still within normal limits. Erythrocytes sedimentate at a rather fair rate of 25 millimetres per hour, which can be considered just mildly abnormal, taking into account the patient’s gender and age. We notice serum creatinine is 140 micromoles per litre, which corresponds, according to Cockroft-Gault, to a creatinine clearance of 67 millilitres per minute. The other chemical constituents and haematological evaluations are unremarkable, except for a rise in the number of neutrophils, which has tripled in comparison with the last examination.

*Impression*

Biochemically, we see little evolution in this slightly overweight, middle-aged patient with a history of arterial hypertension, but there is a possible suspicion of an acute infection. These results need to be correlated to the clinical presentation.

Does this report look familiar? It is unlikely that you have ever received a similar one from your clinical biochemist. Results of biochemical and haematological tests are nearly always presented in a neat, tabular format, with some space for results that cannot be quantified, and for expert comment. In addition, many hospital information systems allow referring clinicians to represent consecutive results graphically, which can help to improve insight into the course of the disease.

## Motivations for structured reporting

If our report does look familiar, it is because it was made according to the same principles that have dominated radiology reporting for over a century. Free text reporting is based on “streams of consciousness”. Radiologists-in-training are taught to look at images in a systematic way and to describe their findings as they see them. At the end of this process, they formulate an impression that summarises significant pathological findings and correlates them with the information and the clinical question provided by the referring physicians. More experienced radiologists make shorter reports [1]. The narrative form of radiology reports, however, is maintained, mainly because we have been trained to do so.

One certainly can question if the way of looking and reasoning of a radiologist has to be the decisive factor when presenting the results of an imaging procedure to the referring physician. Is it essential that he or she can reconstruct our analysis and interpretation? The answer will be “yes” if we suppose the physician will want to personally examine the images, maybe to show and explain the findings to a patient. Such an approach may make sense when talking about examinations with just a few images, such as a chest X-ray or a radiogram of a fracture. It is rather pointless in the case of an ultrasound examination, let alone the hundreds or thousands of images that, for example, result from multislice computed tomography (CT). In such a case, the referring clinician may prefer a nicely structured report, a useful and reproducible instrument for diagnosis and follow-up rather than a roadmap of the mind of the radiologist [2–6]. Moreover, narrative reports do not always address key clinical questions, may contain clinically important errors, may not be transmitted in a timely fashion, and may contain ambiguous terms [7, 8]. In specialty areas such as cardiovascular imaging, policy statements have signalled a move to structured reporting [9]. Other specialty areas which lend themselves rather easily to structured reporting are screening mammography, functional magnetic resonance imaging (fMRI), a number of orthopaedic studies and follow-up studies of oncological patients that use the RECIST criteria.

Reporting imaging studies in a structured way is certainly feasible, as the information in radiology reports is just as amenable to computer-based storage, processing, and displaying as pixels, voxels and images [7].

As David Clunie rightly states, the term “structured reporting” means different things to different people. Simply organising plain text into a preformatted structured document provides only limited benefit. Effective searching and matching also requires the use of “controlled terminology”, chosen from a dictionary of coded concepts, say: a lexicon. A structured report supposes: the presence of lists and hierarchical relationships; the use of coded or numerical content in addition to plain text; the use of relationships between concepts; and the presence of embedded references to images and similar objects [10].

Structured reporting according to these criteria would open up possibilities we can only dream of with our traditional text reports. Automated reasoning could provide radiologists with diagnostic suggestions for unusual cases [7]. In fact, if we consider a structured report as a guideline or as an assistant when reporting, it may well be useful in two ways: for experienced radiologists, as an aid to properly respond to the clinical question, and for inexperienced colleagues as a problem-solver in difficult cases.

Computer-assisted reporting provided by structured reports is facilitated by the possibility to automatically index and retrieve online teaching files or peer-reviewed literature, as well as other retrospective sources of clinical research data—administrative, medical or scientific [7].

## An eternal promise?

Some may believe that structured reporting is the “nuclear fusion reactor” of radiology: an eternal promise, the practical use of which will always be a few decades away. There are huge obstacles to overcome, such as fear of diminished productivity or of less accurate and less complete reports [11, 12]. And indeed, 13 years have passed since the first survey showing a preference for structured reporting among referrers and radiologists, and there is still no sight of the gates of structured reporting heaven.

And yet, we already see their glimmer, for much has been achieved and even more is to come. The collaboration between healthcare providers, the industry and other stakeholders, crystallised in the IHE (Integrating the Healthcare Enterprise) initiative, has resulted in the development and acceptance of a large number of steps forward. Examples of these are Digital Imaging and Communications in Medicine (DICOM) structured reporting, the RSNA Reporting initiative, and the cancer Biomedical Informatics Grid (caBIG) Annotation and Imaging Markup (AIM) project.

## DICOM structured reporting

The DICOM standard has introduced rules for the encoding, transmission and storage of the imaging diagnostic report [8]. The scope of DICOM structured reporting is the standardisation of structured data and clinical observations in the imaging environment [13]. DICOM structured reporting contains text with links to other data such as images, waveforms, and spatial or temporal coordinates. Its structure, along with its wide use of coded information, enables the semantic understanding of the data that is essential for the Electronic Healthcare Record deployment [8]. While DICOM structured reporting provides a data structure allowing to embed structured reports in a standard “container” that can be read across different software applications, it does not define how the content should be structured or standardised. DICOM structured reporting templates have been defined to constrain the possible structures and to provide basic codes that can be used to encode specific reports, such as breast imaging or vascular ultrasound procedures reports.

## Annotation and Image Markup

The AIM project, supported by the U.S. National Cancer Institute’s caBIG initiative, created a standard for adding information and knowledge to an image in a clinical environment. AIM developed a mechanism for modelling, capturing and serialising image annotation and markup data that produces both human- and machine-readable information [14]. AIM has provided a foundation for standardised image annotation practices in the clinical, research and translational communities.

## The need of a standard lexicon for reporting: RadLex

For more than 10 years, RSNA has supported RadLex, which is intended to produce a unifying lexicon for radiology in collaboration with other professional organisations and standards bodies [7, 15]. Representatives of more than 30 radiology professional and standards organisations, including the American College of Radiology (ACR), DICOM and IHE, participated in the process. In November 2006, they publicly released an initial set of more than 7,500 anatomical and pathological terms. In 2007, six additional committees were recruited to develop the RadLex Playbook, which encompasses terms to describe the devices, imaging exams and procedure steps performed in radiology.

With more than 34,000 terms, RadLex satisfies the needs of software developers, system vendors and radiology users by adopting the best features of existing terminology systems, while producing new terms to fill critical gaps. RadLex also provides a comprehensive and technology-friendly replacement for the ACR Index for Radiological Diagnoses. It unifies and supplements other lexicons and standards, such as SNOMED-CT and DICOM. Partial German, Spanish and Portuguese translations are available.

RadLex development has been supported both by the U.S. National Institute of Biomedical Imaging and Bioengineering (NIBIB) and by the U.S. National Cancer Institute’s caBIG project, a large effort to develop a unified computing infrastructure for clinical trials. It is currently being used by RSNA MIRC, myRSNA and reporting committee projects, as well as numerous outside services and applications: commercial reporting and decision support systems, teaching file software, imaging arms of clinical trials, pharmaceutical companies, and professional and standards organisations outside radiology [6, 23]. RSNA has developed a term browser (radlex.org) to give potential users a convenient way to view RadLex’s structure and content. RadLex is available for download via the National Center for Biomedical Ontology (NCBO) BioPortal site (bioportal.bioontology.org).

## Reporting templates, complimentary to RadLex

RSNA has developed a set of templates for structured reporting of radiology results [16]. These templates are based on standard reports from daily practice, submitted by a growing number of radiologists in North America. The templates originally were encoded in the Extensible Markup Language(XML) [17], but the new IHE “Management of Radiology Reporting Templates” (MRRT) format encodes templates using Hypertext Markup Language version 5 (HTML5), code that can be viewed using any web browser [18]. Conversion of templates into the MRRT format is in progress.

RSNA templates are available for download (http://www.radreport.org). The templates themselves are not meant to represent a “standard” for reporting the subjects they pertain to, let alone a tool to force radiologists into one-track thinking. Rather, they are tools which any radiologist or radiology centre can adapt to best suit their needs and insights.

At this moment, more than 200 templates are already online and the number of views has passed the 1 million mark. Hong and Kahn [19] have measured how much of the conventional narrative reports is covered by the concepts included in the RSNA reporting templates. The reporting templates accounted for 17–49 % of the concepts that actually appeared in a sample of corresponding radiology reports. These findings suggest the templates provide useful coverage of the “domain of discourse” in radiology reports.

Structured reports fit perfectly within the framework presented by the European Society of Radiology (ESR) in its communication guidelines for radiologists. Reports should at least be structured into sections on clinical details, technique, imaging findings and conclusion. The ESR guidelines further state that it is not possible to detail the “perfect” report because this will depend on referrer expectation as well as radiologists’ varying opinions, but there is evidence that long free-text reports which do not reach a clear conclusion are those that are least favoured by referrers [20].

At the European Congress of Radiology (ECR) 2013 in Vienna, a collaborative initiative of the RSNA and the European Society of Radiology (ESR) was launched. It is the objective of the RSNA/ESR Reporting Initiative to translate the RSNA templates into a variety of European languages, to adapt them to cover national or specialised applications and to create new templates. A large number of European national societies of radiology as well as specialist and subspecialist societies have expressed support for the initiative, and around 40 of their members have volunteered to collaborate. The RSNA and ESR recently approved a formal agreement for collaboration in the reporting initiative that will allow members of both societies to contribute to the development of a multilingual set of structured report templates.

At the same time, it is useful to look at initiatives which are complementary to those of the RSNA and ESR. One example is the modular approach developed by Fatehi and Safdar [21], which introduces a form of artificial intelligence in the reporting process by interrelating blocks of structured report with the clinical problems presented by the referring clinician. The Society for Interventional Radiology (SIR) has launched a pilot program to capture the information recorded using structured reporting templates into a data registry for aggregate analysis [22]. Similar initiatives worldwide deserve our attention, active interest and involvement.

## Conclusion

Structured reporting clearly has changed from an eternal promise into a device that is ready for deployment. Our “fusion reactor” is ready and it is hungry for fuel!
